# 

**Published:** 1853-04

**Authors:** 


### RECEIPTS

### FOR THE JOURNAL DURING THE MONTH OF MARCH.

ILLINOIS.

| | |
|---|---|
| Dr. Jas. Smitch, | $2 00 |
| " C. J. Miller, | 2 00 |
| " C. F. Folley, | 2 00 |
| " T. C. Patterson, | 2 00 |
| " Julius P. Anthony, | 1 00 |
| " W. T. Luin. | 2 00 |
| " D. Ransom, | 2 00 |
| " L. Lake, | 2 00 |
| " Chas. G. Smith, | 2 00 |
| " B. K. Hart, | 2 00 |
| " L. H. Cutler, | 1 00 |
| " L. T. Leeds, | 2 00 |
| " W. T. Hutchinson, | 2 00 |
| " S. S. Clark, | 2 00 |

MICHIGAN.

| | |
|---|---|
| Dr. A. M. Thomas, | $2 00 |

WISCONSIN.

| | |
|---|---|
| Dr. J. Parker, | $2 00 |
| " Pomeroy & Stough, | 2 00 |
| " R. S. Hayes, | 2 00 |
| " D. L. Downs, | 2 00 |
| " C. A. Hathwell, | 2 00 |
| " W. A. Norman, | 2 00 |
| " H. Truesdail, | 2 00 |
| " Samuel Rifenberrick, | 2 00 |

INDIANA.

| | |
|---|---|
| Dr. R. Q. Wilson, | $2 00 |
| " J. C. Mills, | 3 00 |
| " D. M. Marshall, | 2 00 |
| " T. L. Catherwood, | 2 00 |
| " J. R. Wooden, | 2 00 |

### PROSPECTUS

### OF VOL. II. NEW SERIES OF THE

### NORTH-WESTERN

### MEDICAL AND SURGICAL JOURNAL.

EDITED BY

**W. B. HERRICK, M.D.,**

*Professor of Anatomy and Physiology in Rush Medical College; Surgeon to the U.S. Marine Hospital; one of the Surgeons of the Ill. General Hospital, etc.*

AND

**H. A. JOHNSON, A.M., M.D.**

THE FIRST No. OF VOL. II. OF THE NEW SERIES OF THE NORTH-WESTERN MEDICAL AND SURGICAL JOURNAL, will be published on the 1st of May next.

In order that the volumes hereafter May commence with the year, the present will be published in eight numbers, of 72 pages each, making the same amount of matter as heretofore.

### TERMS:

**TWO DOLLARS PER ANNUM, IN ADVANCE.**

As an inducement to Physicians and others to act as Agents, in extending the circulation of the Journal.

| | | | | |
|---|---|---|---|---|
| Three copies will be furnished to new subscribers for | | | | $5 00 |
| Five | " | " | " | 8 00 |
| Seven | " | " | " | 10 00 |

### TERMS OF ADVERTISING.

| | | | |
|---|---|---|---|
| One page, One year ............ | $20,00 | One page, single insertion........ | $5,00 |
| One-halt page, One year, ...... | 12,00 | One-half page, single insertion.... | 3,00 |

All communications pertaining to the editorial department to be addressed, post-paid, to the "Editors of the N. W. Medical and Surgical Jou nal, Chicago, Ill.;" and all letters on business to "Ballantyne & Co., 63 Clark street, Chicago, Ill."

